# The IL-25/ILC2 axis promotes lung cancer with a concomitant accumulation of immune-suppressive cells in tumors in humans and mice

**DOI:** 10.3389/fimmu.2023.1244437

**Published:** 2023-09-15

**Authors:** Ilham Bahhar, Zeynep Eş, Oğuzhan Köse, Akif Turna, Mehmet Zeki Günlüoğlu, Aslı Çakır, Deniz Duralı, Fay C. Magnusson

**Affiliations:** ^1^ Cancer Research Center, Research Institute for Health Sciences and Technologies (SABITA), Istanbul Medipol University, Istanbul, Türkiye; ^2^ Regenerative and Restorative Medicine Research Center (REMER), Research Institute for Health Sciences and Technologies (SABITA), Istanbul Medipol University, Istanbul, Türkiye; ^3^ Department of Thoracic Surgery, Cerrahpasa Medical School, Istanbul University-Cerrahpasa, Fatih, Istanbul, Türkiye; ^4^ Department of Thoracic Surgery, Faculty of Medicine, Istanbul Medipol University, Istanbul, Türkiye; ^5^ Department of Pathology, Faculty of Medicine, Istanbul Medipol University, Istanbul, Türkiye; ^6^ Department of Medical Microbiology, International School of Medicine, Istanbul Medipol University, Istanbul, Türkiye

**Keywords:** innate lymphoid cells group 2, non-small cell lung cancer, myeloid-derived suppressor cells, regulatory T lymphocytes, innate immunity, adoptive transfer

## Abstract

**Background:**

Group 2 innate lymphoid cells (ILC2) can be activated by interleukin (IL)-33 or IL-25. IL-25-activated ILC2 cells help protect the host against helminth infection while exacerbating allergic-like inflammation and tissue damage in the lung. In the context of cancer, IL-33-activated ILC2 cells were found to bear anti-tumoral functions in lung cancer while IL-25-activated ILC2 cells promoted tumorigenesis in colorectal cancer. The role of IL-25-activated ILC2 cells in lung cancer remains to be addressed.

**Methods:**

We examined the overall survival of human non-small cell lung cancer (NSCLC) patients according to *IL25* expression as well as the distribution of ILC2 cells and regulatory T cells (Tregs) in various NSCLC patient tissues and peripheral blood (PB) of healthy donors (HDs). We analyzed the effect of adoptive transfer of IL-25-activated ILC2 cells on tumor growth, metastasis and survival in a heterotopic murine model of lung cancer.

**Results:**

We report that human NSCLC patients with high *IL-25* expression have reduced overall survival. Moreover, NSCLC patients bear increased frequencies of ILC2s compared to HDs. Frequencies of Tregs were also increased in NSCLC patients, concomitantly with ILC2s. In mice bearing heterotopic lung cancer, adoptive transfer of IL-25-activated ILC2s led to increased tumor growth, increased metastasis and reduced survival. The frequencies of monocytic myeloid-derived suppressor cells (M-MDSCs) were found to be increased in the tumors of mice that received ILC2s as compared to controls.

**Conclusion:**

Overall, our results indicate that the IL-25/ILC2 axis promotes lung cancer potentially by recruiting immune-suppressive cells to the tumors both in humans and in mice, and that it may therefore represent a suitable novel target for NSCLC immunotherapeutic development.

## Introduction

Group 2 innate lymphoid cells (ILC2s) are members of the newly discovered family of innate immune cells that play key roles within barrier tissues. They rapidly respond to tissue insult or injury by releasing interleukin-4 (IL-4), IL-5 and IL-13 and they promote a type 2 adaptive immune response (1). ILC2 cells are known to be enriched in both human and mouse lung, and to be activated by IL-25 and IL-33 (2). The role of ILC2 cells in cancer is highly controversial with some studies showing they are pro-tumorigenic, while others report the opposite (3, 4). In recent years, the number of publications reporting on the role of ILC2 cells in cancer contexts have grown exponentially (4). Nevertheless, a clear role for ILC2 cells in cancer development and metastasis is still not established, underlying how tissue-specific microenvironmental cues may lead to different outcomes. Moreover, recent evidence has called for a distinction between IL-33-activated ILC2 cells and IL-25-activated ILC2 cells as two different subsets of ILC2s with potentially distinct functions (5).

Lung cancer is the leading cause of cancer-related deaths worldwide, with non-small cell lung cancer (NSCLC) being the most common (80%) lung cancer subtype (6). Studies of ILC2 cells in the context of lung cancer are sparse, both in mice and humans. In humans, while ILC2 cells were found to be enriched in NSCLC patients as compared to healthy donors (HDs), suggesting a pro-tumoral role for ILC2 cells (7, 8) seemingly conflicting results were also reported, as ILC2 frequencies were found to be reduced in tumors as compared to normal lung tissue of NSCLC patients (9). The conflicts may be due to differing populations taken as baseline reference, therefore additional and more comprehensive studies comparing the frequencies of ILC2 cells in various tissues of NSCLC patients and HDs are needed.

In mice, IL-33-activated ILC2 cells enhanced anti-tumor immunity in a model for primary and metastatic lung tumor (10). Conversely, they facilitated metastasis to the lung by suppressing natural killer (NK) cells (11). Such opposing functions for IL-33-activated ILC2 cells have also been reported in other cancer types such as models for pancreatic cancer (12). In a mouse model for spontaneous colorectal cancer (CRC), the IL-25-ILC2 axis was found to be pro-tumoral (13), while inhibiting IL-25 signaling or ILC2 cells in an induced model of CRC led to an increased number of tumors (14, 15). These discrepancies highlight the importance of tissue-specific tumor microenvironments as well as their associated ILC2-activating signals when studying the role of ILC2 cells in cancer. To date, the role of IL-25-activated ILC2 cells in a murine model of lung cancer remains unexplored.

Here, we studied the role of the IL-25/ILC2 axis in lung cancer both in humans and in mice. We show that NSCLC patients with high *IL-25* expression have reduced overall survival. We found that ILC2 cells are enriched in NSCLC patients compared to HDs. In NSCLC patients, the increase in ILC2 cells was systemic, resulting in high numbers of ILC2 cells in tumors. Concomitantly with ILC2 cells, we found an increase of immunosuppressive regulatory T cells (Tregs), with a preferential accumulation in tumors and tumor-draining lymph nodes. Adoptive transfer of IL-25-activated ILC2 cells in lung cancer-bearing mice led to increased tumor burden, increased metastasis, and reduced survival. Frequencies of monocytic myeloid-derived suppressor cells (M-MDSCs) were increased in tumors of mice that received ILC2 cells as compared to controls, which may be due to the high production of IL-13 and IL-4 by ILC2 cells. Taken together, our findings highlight a pro-tumoral function for the IL-25/ILC2 axis in NSCLC both in humans and in mice, suggesting that it may constitute a promising therapeutic target in NSCLC.

## Materials and methods

### Human survival analysis

The R2 Genomics Analysis and Visualization Platform (http://r2.amc.nl) was used for the Kaplan-Meier overall survival curve for patients with high and low *IL-25* expression. The ‘Mixed Lung Adenocarcinoma TCGA 2022 v32’ dataset was used for visualization with the Kaplan Scan tool of the R2 Genomics Analysis and Visualization Platform, determining the optimal survival cut-off based on statistical tests within the web application.

### Patients and healthy donors

Peripheral blood, tumor sample, along with an adjacent non-invaded tissue sample and mediastinal lymph node sample were collected from 30 patients with NSCLC undergoing surgical resection at Cerrahpasa Hospital and Medipol Mega Hospital in Istanbul. Prior to sampling, none of the patients had received chemotherapy, radiation therapy or immunotherapy. All subjects gave written informed consent to participate in this study. The study protocol was approved by Istanbul Medipol University Ethics Committee. The study was conducted in accordance with the ethical guidelines of the Declaration of Helsinki. Details of the clinicopathological characteristics of these patients are summarized in **Supplementary Table S1**. As a control, peripheral blood samples were taken from 14 sex- and age-matched healthy donors (HD).

### Human cell isolation

Peripheral blood mononuclear cells (PBMCs) were obtained from blood samples by the Lymphocytes Separation Media (cat. no. LSM-A; Capricorn, Germany) density gradient centrifugation method. Briefly, whole blood was diluted by the addition of an equal volume of PBS, blotted slowly onto separation medium, and centrifuged at 2000 rpm for 20 minutes at room temperature without stopping or acceleration. Cells were harvested from the interface, washed twice in phosphate buffered saline (PBS), then counted and prepared for staining. Tumor tissues and adjacent noncancerous tissues were dissociated into single cell suspensions using the human tumor dissociation kit (cat. no. 130-095-929; Miltenyi, Germany) according to the manufacturer’s instructions. Mediastinal lymph node tissues were mechanically dissociated into single cell suspensions. Red blood cells were removed from single cell suspensions using an ammonium chloride solution prepared in-house. The cells were filtered using a 70 µm cell strainer and then washed with PBS+ 2% fetal bovine serum (FBS) for subsequent flow cytometric assays.

### Mice

Wild-type (WT) C57BL/6J mice were provided by Medipol University Medical Research Center (Istanbul, Turkey). B6PL-Thy1.1 mice were obtained from The Jackson Laboratory (Bar Harbor, ME). Six- to eight-week-old C57BL/6J mice of both sexes were used as recipients of LLc1 tumor cells (tumor-bearing mice). B6PL-Thy1.1 mice of both sexes, six to eight weeks old, were used as donor mice for ILC2 isolation. For animal experiments, mice of the same age and sex were randomly assigned to experimental groups. Mice were housed under controlled conditions of temperatures of ~18-23°C with 40-60% humidity and a 12/12 h reverse light/dark cycle. Mouse care and experimental procedures were performed according to federal guidelines and protocols approved by Istanbul Medipol University Animal Experiments Local Ethics Committee (IMU-HADYEK) with protocol number 43. Tumor-bearing mice were monitored three times a week and at shorter intervals depending on the condition of the mice. Mice showing signs of stress, discomfort, pain, lethargy, inability to properly groom themselves, or inability to obtain food and/or water were immediately sacrificed.

### Tumor cell line

The murine Lewis lung carcinoma (LLc1) cell line was kindly provided by Prof. Dr. Güneş Esendağlı (Hacettepe University-Ankara/Turkey) at passage number 6 from the original stock obtained from ATCC (CRL-1642). The cells were tested negative for mycoplasma with the EZ-PCR Mycoplasma Detection kit (Biological Industries, cat. 20-700-20). Upon receipt, the cells were expanded, and aliquots were frozen to allow use of low passage cells throughout the study. Thawed cells were maintained in RPMI 1640 medium (Sigma-Aldrich) supplemented with 2 mM L-glutamine, 1 mM sodium pyruvate, 10 mM HEPES, non-essential amino acids (Sigma-Aldrich), 10% FBS and 1% penicillin–streptomycin (Gibco) (complete RPMI medium) at 37°C in a humidified atmosphere with 5% CO_2_. Before injection, LLc1 cells (70–80% confluency) were harvested. Briefly, the cells were washed with PBS and detached using 0.25% trypsin-EDTA. Trypsin was neutralized with medium containing 10% FBS. After centrifugation, the cells were resuspended in PBS.

### Adoptive transfer of ILC2s

Briefly, for *in vivo* induction of ILC2s, donor mice (B6PL-Thy1.1) were hydrodynamically injected with 10 µg of a plasmid encoding murine *IL25* (pCMV3-mIL25, Sino Biological) as previously described (16). Mice were sacrificed three days post-injection, spleens and mesenteric lymph nodes (MLN) were harvested and mechanically dissociated into single cell suspensions. Red blood cells from spleen single cell suspensions were lysed using an ammonium chloride solution prepared in-house. MLN and spleen single cell suspensions were filtered through a 70µm cell strainer and washed with PBS+2% FBS. Lineage-positive cells were depleted from the resulting cell suspensions using the mouse direct lineage cell depletion kit (cat. 130-110-470, Miltenyi Biotec) according to the manufacturer’s instructions. The flow through containing lineage negative (Lin-) cells was washed with PBS+2% FBS before antibody staining and fluorescence-activated cell sorting (FACS) for CD45+ Lin- ICOS+ KLRG1+ ILC2s. FACS purified ILC2s were washed in PBS before injecting intravenously (i.v.) via the tail vein into tumor-bearing C57BL/6J recipient mice. Each injection consisted of 500,000 ILC2s in PBS. Each mouse received 3 injections per week for 4 weeks, and tissues were collected 5 days after the final injection for assessment of immune cell composition, which included 5 and 6 mice per group. For the survival experiment (5 and 4 mice per group), mice continued to be monitored after the final ILC2 injection until natural death or euthanasia. Mice were euthanized when reaching humane endpoint (showing clinical signs of stress, discomfort, pain, lethargy, inability to properly groom themselves, inability to obtain food and/or water, hunching, weight loss above 20% of body weight, or a tumor volume above 1000mm^3^). For the tumor burden experiment, a total of 10 and 9 mice per group pooled from 3 independent experiments were assessed. For the experiment assessing the migration of ILC2s which included 3 mice per group, each mouse received a single injection of ILC2s, and tissues were collected 24h later. All ILC2s for adoptive transfers were prepared fresh using the above protocol.

### Mouse lymphodepletion

Tumor-bearing WT C57BL/6J mice were partially lymphodepleted by injecting cyclophosphamide (CTX, Sigma-Aldrich) intraperitoneally (i.p.) as a single dose at 200 mg/kg.

### Tumor volume assessment

6 - 8-week-old WT C57BL/6J mice were inoculated subcutaneously (s.c.) in the axilla area of the right forelimb with 1x10^5^ LLc1 cells in 100 µl sterile PBS per mouse. Tumor growth was assessed three times per week by measuring tumor dimensions with calipers. Tumor volume was calculated using the following formula: V = (W^2^ × L)/2, W= Width; L= Length.

### Metastasis assessment

At the time of natural death or euthanasia as indicated in ‘adoptive transfer of ILC2 cells’, left and right lobes of the lungs were collected from mice (a total of 9 and 6 mice per group pooled from 2 independent experiments) and immediately placed separately in fixative solution (10% buffered formaldehyde). Fixed lung tissues were then embedded in paraffin. Paraffin blocks were then sliced into 4µm sections, which were stained with hematoxylin and eosin (H&E). Images from whole left and right lobe sections were taken using the Zeiss Axio Zoom V16 confocal microscope with Plan-NeoFluar Z objective set on brightfield mode. Tumor and non-tumor regions were manually defined on images taken from the left and right lobes using QuPath software (version 0.4.3). Percent lung metastatic area was calculated by dividing tumor lung area by total lung tissue area x 100 using QuPath software. Number of tumor nodules were manually counted.

### Flow cytometry

For surface staining, both human and murine single cell suspensions were first stained with a viability marker (Fixable Viability Kit, Biolegend) according to manufacturer’s instructions to exclude dead cells, then Fc gamma receptors were blocked for 15 minutes with heat-inactivated human serum prepared in-house or anti-mouse CD16/32 (TruStain FcX, Biolegend) for human samples and mouse samples respectively, in PBS+2% FBS, and then incubated with the specific fluorochrome-labeled antibodies (all purchased from Biolegend unless otherwise noted) for 20 minutes at 4°C in the dark in PBS+2% FBS. For intracellular transcription factor staining, cells were fixed and permeabilized using a True-Nuclear Transcription Factor buffer kit (Biolegend) for 30 minutes at 4°C in the dark, washed twice with Perm buffer, and then stained for 30 minutes in Perm buffer. For intracellular cytokine staining on murine *in vivo*-induced ILC2 cells, freshly sorted cells were stained ex vivo or after stimulation with carrier-free recombinant mouse IL25 (Biolegend) at a concentration of 100 ng/ml in complete RPMI medium for 36 h. Both ex vivo and IL25-stimulated cells were cultured in complete RPMI medium in the presence of phorbol-12-myristate-13-acetate (500 ng/ml) (Sigma-Aldrich) and ionomycin (250 ng/ml) (Sigma-Aldrich) for 4 h at 37°C immediately prior to staining. Brefeldin A (Biolegend) was added in the last 3 h of the culture. Cells were then surface stained as indicated above. Cells were then fixed, permeabilized and stained using True-Nuclear Transcription Factor buffer kit (Biolegend) according to manufacturer’s instructions. After staining, cells were washed with PBS+2% FBS, then resuspended in PBS+2% FBS for acquisition on a flow cytometer. Acquisition of human samples was performed on BD Influx. Acquisition of murine samples was performed on BD Symphony A1. Analyses of acquired data were performed using Flow Jo v10.9. Cell sorting was performed using BD Influx with >98% purity.

### Antibodies

For human samples, single-cell suspensions were stained with Zombie UV fixable viability kit (Biolegend), FITC-conjugated Hematopoietic Lineage cocktail prepared in-house (CD2 (TS1/8), CD3 (UCHT1), CD11b (ICRF44), CD14 (HCD14), CD15(HI98), CD16 (3G8), CD19 (HIB19), CD34 (561), CD56 (HCD56), CD123(6H6), CD20 (2H7), FcϵRIα AER-37 (CRA-1)), PE-conjugated anti-CD127 (A019D5), APC/Cy7–conjugated anti-CD45 (2D1)), APC-conjugated anti-CD294 (CRTH2) (BM16), PE/Cy7-conjugated anti-HLA-DR (L243), BV421-conjugated anti-CD86 (BU63), BV510-conjugated anti-PD-L1 (29E.2A3), PE/Dazzle™ 594-conjugated anti-CD25 (BC96) and BUV661-conjugated anti-CD4 (SK3) or appropriate isotype controls.

For mouse experiments, the following antibodies were used for surface staining of ILC2 cells: FITC-conjugated Lineage cocktail prepared in-house (anti-CD3 (17A2), anti-CD5 (53-7.3), anti-B220 (RA3-6B2), anti-CD11b (M1/70), anti-CD11c (N418), anti-NK1.1 (PK136), anti-TER-119 (TER-119), anti-Gr1 (RB6-8C5), anti-CD170 (S17007L), anti-FcϵRIα (Mar.01), anti-CD19 (6D5), anti-TCRβ (H57-597), anti-TCRγ/δ (UC7-13D5), PE-conjugated anti-CD45 (30.F11), APC-conjugated anti-KLRG (2F1/KLRG1), PE/Cy7 conjugated anti-CD278 (ICOS) (C398.4A), PerCP/Cyanine5.5-conjugated anti-CD90.1 (Thy1.1) (OX-7), Zombie UV fixable viability dye. For intracellular staining of ILC2 cells, the following antibodies were used: PE-conjugated anti-IL-5 (TRFK5), APC/Cy7-conjugated anti-IL-13 (eBioscience, clone eBio13A), PE/Dazzle 594-conjugated anti-IL-4 (11B11), and PE/Cy7-conjugated anti-GATA3 (eBioscience, clone TWAJ) or appropriate isotype controls. For immune cell composition of tissues obtained from tumor-bearing mice, the following antibodies were used for surface staining: PE/Cy7-conjugated anti-CD3 (eBioscience, clone 145-2C11), BV785-conjugated anti-CD4 (GK1.5), BV510-conjugated anti-CD8 (GK1.5), AF594-conjugated anti-CD11b (M1/70), FITC-conjugated anti-CD11c (N418), PE-conjugated anti-Ly6C (HK1.4), APC-conjugated anti-Ly6G (1A8), PE/Cy5-conjugated anti-MHCII (M5/114.15.2), Zombie UV fixable viability dye. For intracellular staining, AF700-conjugated anti-FOXP3 (MF-14) was used.

### Murine cell isolation

A maximum of 1g of tumor and lung tissue was rinsed with PBS, cut into small pieces using scissors and placed in a gentleMACS C tube (Miltenyi Biotec) in 5 ml RPMI1640 containing 100 µl 10X collagenase/hyaluronidase enzyme mixture (cat. 07912, Stemcell). The C tubes were then placed on a gentleMACS dissociator (Miltenyi Biotec) and the appropriate programs were run according to the mouse tumor dissociation kit protocol and mouse lung dissociation kit protocol (both from Miltenyi Biotec) for tumors and lungs respectively. The samples were then incubated for 45 min at 37°C while rotating. The digested fragments were manually dissociated further by mashing them with a syringe plunger onto a 70 µm cell strainer and the cells were washed in PBS+ 2% FBS. Erythrocytes were removed with an ammonium chloride solution prepared in-house. Mononuclear leukocytes from tumors were isolated by performing a Histopaque 1083 (Sigma-Aldrich) density gradient centrifugation according to the manufacturer’s instructions. Obtained single cell suspensions were then washed in cold PBS+ 2% FBS buffer before staining. Liver tissue was dissociated by following the ‘preparation of single cell suspensions from mouse liver’ protocol from Miltenyi Biotec. Cells were then washed in cold PBS+ 2% FBS buffer before staining. Spleen and lymph nodes were mechanically dissociated then filtered through a 70-µm cell strainer. Red blood cells from the spleen were removed using ammonium chloride solution prepared in-house. Samples were washed in cold PBS+ 2% FBS buffer prior to staining.

### Statistical analysis

Statistical analyses and representations were performed using Prism (GraphPad Version 9.0) and are detailed in the figure legends.

## Results

### IL-25 is associated with reduced survival while ILC2 cells and Tregs are enriched in NSCLC patients

**Figure 1:**
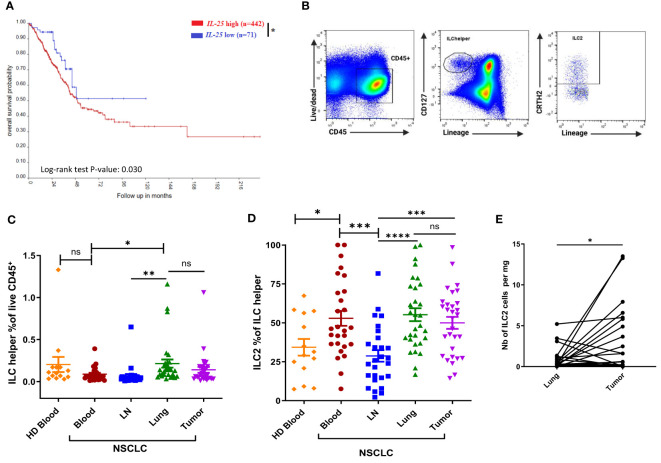
Association between *IL25* expression and NSCLC patient overall survival, and distribution of ILC2 cells in human NSCLC tissues and HDs. **(A)** Kaplan-Meier curve of high or low *IL25* expression, n indicates the number of patients in each group. R2 bioinformatics platform was used to probe available TCGA data on NSCLC. **(B)** Representative gating strategies for total ILC helpers (live CD45^+^ Lin (CD2, CD3, CD11b, CD14, CD15, CD16, CD19, CD34, CD56, CD123, CD20, FcϵRIα)^-^ CD127^+^) and ILC2 cells (live CD45^+^ Lin^-^ CD127^+^ CRTH2^+^). **(C)** Frequencies of total ILC helpers among CD45^+^ cells in tissues obtained from NSCLC patients (Blood and LN, n=27; Tumor and Lung, n=30) and PB obtained from HD (n=14). **(D)** Percentages of ILC2s among total ILC helpers in NSCLC tissues (Blood and LN, n=27; Tumor and Lung, n=30) and PB from HD (n=14). **(E)** Absolute numbers of ILC2 cells per mg of tissue in tumor tissue and matched normal lung tissue obtained from NSCLC patients (for each group, n=30). LN: tumor-draining mediastinal lymph node; Lung: normal lung tissue adjacent to tumor tissue; HD: healthy donor. ns, not significant; *:p<0.05; **:p<0.01; ***:p<0.001; ****p<0.0001. P-values were obtained in a log-rank test **(A)**, an unpaired Student’s t-test **(B–D)** and paired Student’s t-test **(E)**. Horizontal bars mark mean. Error bars mark standard error of mean (sem).

We examined the survival probability of NSCLC patients with high and low *IL25* gene expression by applying the R2 Genomics Analysis and Visualization Platform to the large publicly available TCGA dataset. Patients with high *IL25* expression as determined by the Kaplan Scan tool were found to have significantly reduced overall survival (p-value 0.03) as compared to *IL25*-low patients (**Figure 1A**). IL-25 being a known activator of ILC2 cells, we sought to determine the frequencies of ILC2 cells in NSCLC patients. We obtained fresh surgically resected human primary NSCLC tumors, as well as adjacent normal lung tissue, tumor-draining mediastinal lymph nodes (LNs) and peripheral blood (PB) from the same patients. As a control, we also obtained PB from HDs. All samples from NSCLC patients and HDs were analyzed for their relative abundance in total ILC helpers as well as ILC2 cells by flow cytometry. Total helper ILCs were identified as live CD45^+^ Lin^-^ CD127^+^ cells, among which ILC2 cells were identified as CRTH2^+^ cells, in line with the literature and previous studies (1, 8, 9). Representative gating strategies for total ILC helpers and ILC2 cells are shown in **Figure 1B**.

As shown in **Figure 1C**, there was no significant difference between the frequencies of circulating total ILC helpers in NSCLC patients and HDs. No substantial difference in the frequency of total ILC helpers was observed between tumors and their normal counterparts (**Figure 1C**). Of note, ILC helpers were found to be more highly represented in patients’ lungs than in their LNs or PB (**Figure 1C**), consistent with the known tissue distribution of ILCs (1, 17). Next, we compared the relative abundance of ILC2 cells within total ILC helpers (**Figure 1D**). NSCLC patients had significantly higher frequencies of circulating ILC2 cells as compared to HDs (**Figure 1D**). There was no significant difference between the frequencies of ILC2 cells in tumors and their normal counterparts or patient PB (**Figure 1D**). Of note, ILC2 cells were less represented in mediastinal LNs compared to other tissues (**Figure 1D**), consistent with the known tissue distribution of ILC2s (1, 17). Calculation of ILC2 absolute numbers per mg of tissue revealed higher numbers of ILC2 cells in tumors than their normal counterparts (**Figure 1E**).

Murine ILC2 cells have been reported to express MHC Class II and to be able to function as antigen-presenting cells (APCs) (18–21). In humans, only one study reported MHC Class II and CD80 expression on ILC2 cells as being elevated in patients with acute exacerbation of chronic pulmonary obstructive pulmonary disease (AECOPD) (22). Moreover, ILC2 cells stimulated by IL-33 were found to upregulate PD-L1 (23). PD-L1 is a ligand for PD-1, which is a known checkpoint inhibitor of CD4^+^ T cells that plays important roles in diminishing anti-tumor immunity. We therefore wanted to assess whether ILC2 cells in NSCLC patients may have immunosuppressive capacities by interacting directly with CD4^+^ T cells via MHC II, CD86 and PD-L1. To this end, we assessed the expression of HLA-DR, CD86 and PD-L1 on ILC2 cells in the various tissues obtained from NSCLC patients as well as in HDs (**Supplementary Figure 1**). ILC2 cells in PB of HDs and tumors of NSCLC patients did not express HLA-DR, CD86 nor PD-L1 (**Supplementary Figure 1**). In NSCLC patients, the lack of expression of HLA-DR, CD86 and PD-L1 by ILC2 cells was irrespective of the tissue of origin (data not shown). ILC2 cells from NSCLC and HD samples expressed CD25 (**Supplementary Figure 1**), consistent with the literature (24). CD25 expression by ILC2 cells in samples obtained from NSCLC patients was irrespective of the tissue of origin (data not shown).

**Figure 2:**
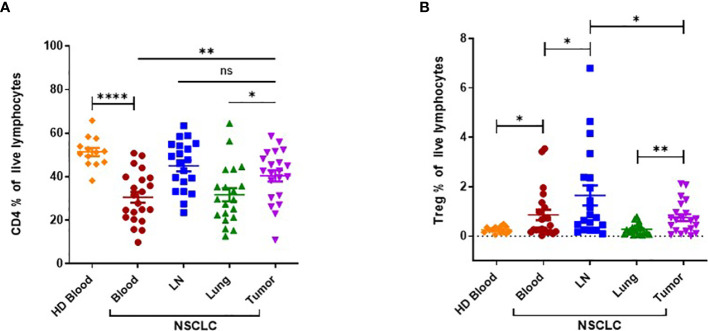
Flow cytometric analysis of CD4^+^ T cells and CD4^+^ CD25^hi^ regulatory T cells (Tregs) in NSCLC patients and HDs. **(A)** Frequency of CD4^+^ T cells in tissues obtained from NSCLC patients (peripheral blood (PB), n=22; LN, n=20; Lung; n=20; Tumor, n=21) and PB obtained from HDs (n=14). **(B)** Frequency of CD4^+^ CD25^hi^ Tregs in tissues obtained from NSCLC patients (PB, n=22; LN, n=20; Lung; n=20; Tumor, n=21) and PB obtained from HDs (n=14). LN: tumor-draining mediastinal lymph node; Lung: normal lung tissue adjacent to tumor tissue; HD: healthy donor. Horizontal bars mark mean. Error bars mark sem. P-values were calculated with unpaired Student’s t-test. *p < 0.05; **p < 0.01; ****p < 0.0001; ns, not significant.

Next, we assessed the frequencies of total CD4^+^ T lymphocytes and CD4^+^ CD25^hi^ Tregs by flow cytometry (**Figure 2**). Circulating CD4^+^ T cells were present in significantly lower amount in NSCLC patients compared to HDs (**Figure 2A**). The frequency of CD4^+^ T cells was significantly higher in tumors than in matched normal lung tissues or patient PB. There was no significant difference between frequencies of CD4^+^ T cells in tumors and LNs. Circulating Treg frequencies were found to be significantly elevated in NSCLC patients compared to HDs (**Figure 2B**). Tumor-infiltrating Tregs were present in significantly higher amount if compared with normal tissues. Tregs were represented the highest in LNs.

In summary, we report that high *IL25* expression in NSCLC patients is associated with reduced survival. NSCLC patients bear a systemic increase of ILC2 cells, resulting in high numbers of ILC2 cells in tumors. Frequencies of Tregs are also increased in patients compared to HDs, with a preferential accumulation in tumors and tumor-draining LNs. Taken together, these findings suggest that the IL25/ILC2 axis and Tregs may concomitantly contribute to a tumor-promoting environment in human NSCLC.

### Adoptive transfer of IL-25-activated ILC2 cells in tumor-bearing mice promotes tumor growth and metastasis, and reduces survival

In order to test our hypothesis that the IL25/ILC2 axis bears tumor-promoting properties, we decided to examine the effect of adoptive transfer of IL25-activated ILC2 cells in lung tumor-bearing mice using the well-established Lewis lung carcinoma (LLc1) heterotopic model of lung cancer. The LLc1 tumor cell line is known to be highly tumorigenic but weakly metastatic in mice.

**Figure 3:**
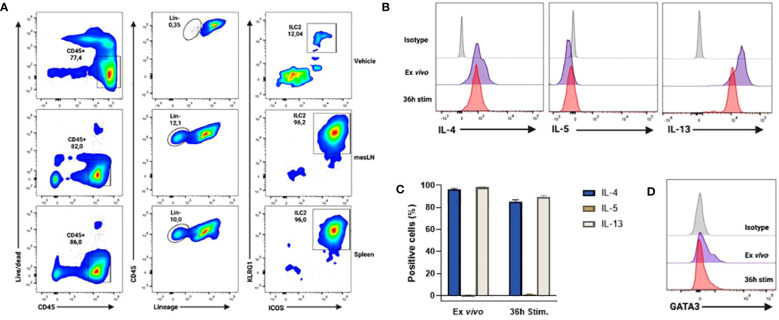
Phenotype and cytokine profile of *in vivo* expanded ILC2s after hydrodynamic gene delivery (hgd) with pCMV-IL25 in mice. **(A)** Gating strategy for the identification of ILC2 cells (live CD45^+^ Lin (CD3, CD5, B220, CD11b, CD11c, NK1.1, TER-119, Gr1, CD170, FcϵRIα, CD19, TCRβ, TCRγ/δ)^-^ KLRG1^+^ ICOS^+^) 3 days after hgd with vehicle in spleen (top panel) or after hgd with pCMV-IL25 (bottom 2 panels) in mesLN and spleen of B6PL-Thy1.1 mice. mesLN: mesenteric lymph node. **(B)** Intracellular cytokine analysis of *in vivo* expanded ILC2 cells from mesLN, stimulated with PMA/ionomycin for 4h directly after obtention of mononuclear cell suspension (ex vivo, purple line) or after 36h restimulation with murine recombinant IL-25 (36h stim, red line). Results are shown as gated on live Lin- ICOS^+^ cells. Appropriate isotype controls (gray lines) are shown. One representative example out of 3 is shown. **(C)** Pooled data (n=3) are shown. Bars represent the mean values of the percentages of positive cells ± sem. **(D)** Representative example of GATA3 transcription factor expression by *in vivo* expanded ILC2 cells stained directly after obtention of mononuclear cell suspension (ex vivo purple line) or after 36h restimulation with murine recombinant IL-25 (36h stim, red line) as stated in **(B)** Appropriate isotype (gray line) is used as control. One representative example out of 3 is shown.

The obtention of IL25-activated ILC2 cells for adoptive transfer purposes was achieved by endogenous ILC2 cell expansion *in vivo* by hydrodynamic gene delivery (hgd) of an IL25-encoding plasmid (pCMV-IL25) as previously described (16). Such ILC2 cells were identified as CD45^+^ Lin^-^ KLRG1^+^ ICOS^+^ cells and were recovered from mesenteric LNs (MLNs) and spleen as previously described (16). A representative example of the *in vivo* expansion and phenotypic profile of such ILC2 cells following hgd is shown in **Figure 3A**. These cells were high producers of IL-4 and IL-13, but not IL-5 (**Figures 3B, C**). Their cytokine production profile remained the same when cells were restimulated with murine IL-25 *in vitro* (**Figures 3B, C**). Moreover, these cells expressed the master transcription factor GATA3 (**Figure 3D**). Altogether, these results show that CD45^+^ Lin^-^ KLRG1^+^ ICOS^+^ cells obtained after hgd with IL-25-encoding plasmid were true ILC2 cells that expressed type 2 cytokines and GATA3. Moreover, their expansion by IL-25 and high KLRG1 expression reminisce of the inflammatory subset of ILC2 cells (iILC2) previously described (5).

**Figure 4:**
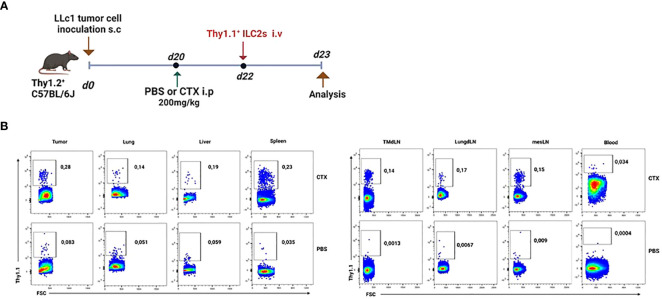
Migration of adoptively-transferred ILC2 cells in tumor-bearing mice. **(A)** Schematic representation of experimental design. Tumor-bearing Thy1.2^+^ C57BL/6J mice were partially lymphodepleted by receiving CTX at 200mg/kg in PBS i.p. Controls received PBS only. 48h later, mice were adoptively transferred with 5x10^5^ Thy1.1^+^ ILC2 cells i.v. in the tail vein. Recipient mice were sacrificed 24h after adoptive transfer and tissues were collected. i.p: intraperitoneal; i.v.: intravenous. **(B)** Representative flow cytometry plots showing the percentage of adoptively-transferred Thy1.1^+^ ILC2s in various tissues obtained from the lymphodepleted (CTX, upper panels, n=3) Thy1.2^+^ tumor-bearing recipient mice and non-lymphodepleted (PBS, lower panels, n=3) recipient mice. Plots are gated on live lymphocytes.

In order to temporarily free up niches for adoptively transferred cells, we decided to partially lymphodeplete recipient mice by administering cyclophosphamide (CTX) i.p. one day prior to adoptive transfer. First, we wanted to assess the migration potential of IL25-activated ILC2 cells adoptively transferred i.v. into tumor-bearing mice. To this end, we transferred Thy1.1^+^ ILC2 cells into tumor-bearing Thy1.2^+^ recipient mice two days after CTX or PBS vehicle injection (**Figure 4A**). The recipient mice were sacrificed 24h after ILC2 transfer and various organs were collected for staining with anti-Thy1.1 antibody (**Figure 4A**). As shown in **Figure 4B**, transferred IL-25-activated ILC2 cells migrated to all tissues of recipient mice, including tumors. In mice that were not lymphodepleted prior to ILC2 adoptive transfer, the percentage of transferred cells was very low in all tissues, and almost undetectable in PB and secondary lymphoid organs (**Figure 4B**, lower panels). Lymphodepletion with CTX prior to adoptive transfer enabled a 3-fold increase in the frequency of transferred cells that could be recovered in tumors (**Figure 4B**, 0.083% in vehicle-treated mice vs. 0.28% in CTX-treated mice).

**Figure 5:**
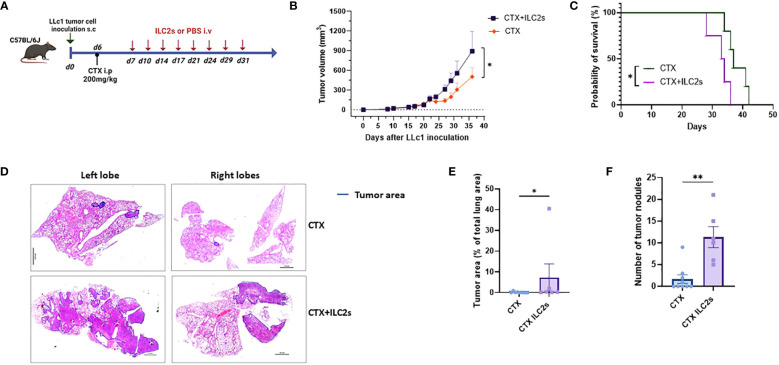
Adoptive transfer of ILC2 cells increases tumor burden and metastasis, and decreases survival. **(A)** Schematic representation of experimental design. 6-8-week old C56BL/6J mice were inoculated with 1x10^5^ LLc1 lung tumor cells on day 0, then partially lymphodepleted with CTX on day 6. Starting one day after CTX administration, mice received 5x10^5^ freshly sorted ILC2 cells or vehicle 2 times per week for 4 weeks. s.c: subcutaneous; i.p: intraperitoneal; i.v.: intravenous. **(B)** Tumor volume in mice that received ILC2s (CTX+ILC2s; n=9) vs. mice that received vehicle (CTX; n=10). Data show mean ± sem. Data were pooled from 3 independent experiments with ≥3 mice per group. **(C)** Survival of mice mice that received ILC2s (CTX+ILC2s; n=4) vs. mice that received vehicle (CTX; n=5). **(D)** Representative examples of metastasis identification (encircled in blue) in images of H&E-stained sections from lungs of mice that received ILC2s (CTX+ILC2s) vs lungs of mice that received vehicle (CTX). Scale bar represents 100 pixel in each image. **(E)** Percentage of tumor area among total lung area in lungs of mice that received ILC2s (CTX+ILC2s; n=6) vs lungs of mice that received vehicle (CTX; n=9). **(F)** Number of tumor nodules in total lungs of mice that received ILC2s (CTX+ILC2s; n=6) vs lungs of mice that received vehicle (CTX; n=9). *:p<0.05. **:p<0.01. p values were determined by Student’s t-test **(B, E, F)** and log-rank test **(C)**.

Next, in order to assess the effect of IL25-activated ILC2 cells on tumor growth, metastasis to the lung and overall survival, we transferred freshly sorted ILC2 cells or vehicle into lymphodepleted tumor-bearing mice twice per week for 4 weeks (**Figure 5A**). The purity of ILC2 cells after cell sorting was at or above 98% (**Supplementary Figure 2**). Adoptive transfer of ILC2 cells had no effect on tumor growth in mice that did not receive CTX treatment (**Supplementary Figure 3**). This is probably due to the fact that only very low numbers of ILC2 cells could reach the tumor in non-lymphodepleted tumor-bearing mice as stated above. Although initially the tumor volume was similar in mice that received IL25-activated ILC2 cells and controls, it started to become significantly higher in mice that received the cells as compared to those that received vehicle after the second week of treatment (**Figure 5B**). Overall, IL25-activated ILC2 adoptive transfer led to significant higher tumor burden (**Figure 5B**). Moreover, the survival of mice was significantly reduced when IL25-activated ILC2 cells were transferred (**Figure 5C**). Metastasis to the lung was assessed in CTX-treated mice that received IL25-activated ILC2 cells and those that received vehicle. Transfer of IL25-activated ILC2 cells led to a significantly higher metastasis burden both in overall percentage of tumor area and number of tumor nodules (**Figures 5D–F**).

Taken together, these results indicate that IL25-activated ILC2 cells promote tumor growth, increase the metastatic potential of tumor cells, and reduce the survival of tumor-bearing mice.

### Adoptive transfer of IL25-activated ILC2 cells leads to accumulation of suppressive M-MDSCs into tumors

**Figure 6:**
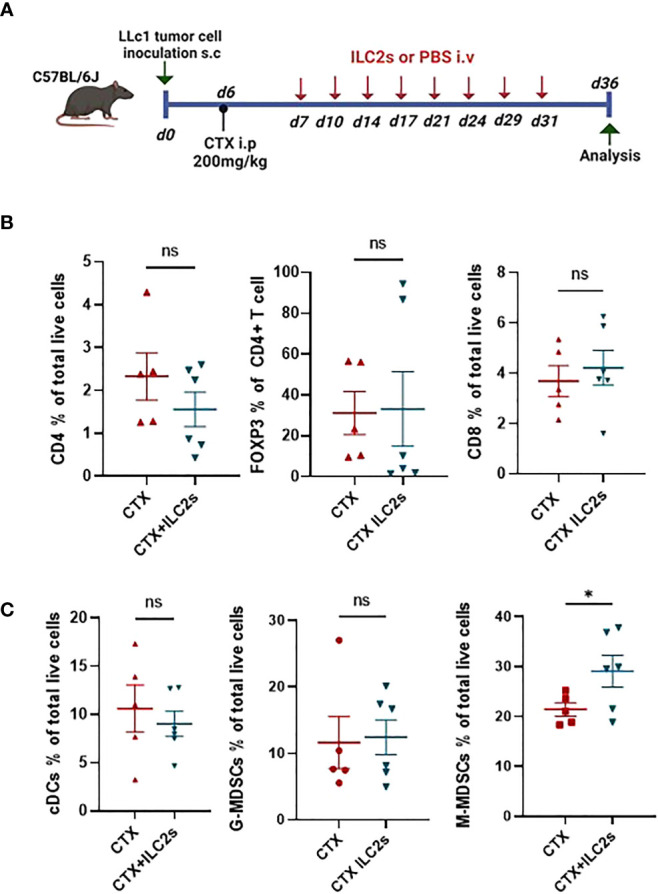
Adoptive transfer of ILC2 cells promotes the accumulation of M-MDSCs in tumors. **(A)** Schematic representation of experimental design. 6-8-week old C56BL/6J mice were inoculated with 1x10^5^ LLc1 lung tumor cells on day 0, then partially lymphodepleted with CTX on day 6. Starting one day after CTX administration, mice received 5x10^5^ freshly sorted ILC2 cells or vehicle 2 times per week for 4 weeks. Mice were sacrificed 5 days after the last injection and tumors were collected. s.c: subcutaneous; i.p: intraperitoneal; i.v.: intravenous. **(B)** Frequency of tumor-infiltrating CD4 T cells identified as CD3^+^ CD4^+^, CD8 T cells identified as CD3^+^ CD8^+^ and CD4 regulatory T (Treg) cells identified as CD3^+^ CD4^+^ Foxp3^+^ in vehicle (CTX, n=5)- and ILC2-treated (CTX+ILC2, n=6) tumor-bearing mice. **(C)** Frequency of tumor-infiltrating conventional DCs (cDCs) identified as CD11c^+^ MHCII^+^ cells, granulocytic myeloid-derived suppressor cells (G-MDSCs) identified as CD3^-^ CD11b^+^ Ly6G^+^ Ly6C^low^, and monocytic MDSCs (M-MDSCs) identified as CD3^-^ CD11b^+^ Ly6G^-^ Ly6C^hi^ in vehicle (CTX, n=5)- and ILC2-treated (CTX+ILC2, n=6) tumor-bearing mice. Horizontal bars mark mean. Error bars mark sem. Statistical significance was calculated by unpaired two-tailed t-test. ns, not significant; *:p<0.05.

Next, we sought to determine the TME composition after IL25-activated ILC2 transfer in CTX-lymphodepleted tumor-bearing mice. To this end, we transferred freshly sorted ILC2 cells or vehicle into lymphodepleted tumor-bearing mice twice per week for 4 weeks. Recipient mice were sacrificed 5 days after the last ILC2 injection and tumors, spleens and lymph nodes (LNs) were collected for analysis (**Figure 6A**). As shown in **Figure 6B**, the percentages of CD4^+^, CD8^+^ and Foxp3^+^ Treg T lymphocyte subsets were similar in tumors of mice that received ILC2 cells or PBS. **Figure 6C** shows the percentages of myeloid cells in tumors. IL25-activated ILC2 cell transfer did not alter the frequencies of conventional dendritic cells (cDCs) nor granulocytic MDSCs (G-MDSCs), however there was a significant increase in the frequencies of monocytic MDSCs (M-MDSCs) in mice that received IL25-activated ILC2 cells as compared to controls (**Figure 6C**). This is in line with a previous report on IL-25-activated ILC2 cells promoting colorectal cancer by activating M-MDSCs (13). The accumulation of M-MDSCs in tumor-bearing mice that received IL25-activated ILC2 cells was not systemic but rather specific to tumors since there was no significant difference in their frequencies within spleens and LNs (**Supplementary Figure 4** and data not shown). This result is consistent with the higher migration potential of IL25-activated ILC2 cells into tumors as compared to other tissues (**Figure 4B**).

Overall, our results show that IL25-activated ILC2 cells preferentially migrate to tumors, and that they promote tumor growth and metastasis, as well as reduce survival of tumor-bearing mice. Moreover, we found that M-MDSCs accumulated into tumors following adoptive transfer with IL25-activated ILC2 cells, suggesting that these cells may actively recruit M-MDSCs into tumors. This may be due to the high production of IL-4 and IL-13 by IL25-activated ILC2 cells (**Figures 3B, C**) as it has previously been shown that ILC2s mediate the recruitment of MDSCs via IL-13 (25, 26) and that they induce the expansion and activation of M-MDSCs via the activation of STAT6 through the binding of the IL4Ralpha (13, 27).

## Discussion

Here, we found that *IL25* expression is negatively correlated with survival of human NSCLC patients, that ILC2 cells are elevated in human NSCLC patients and that IL25-activated murine ILC2 cells promote lung tumor growth and metastasis while reducing survival of tumor-bearing mice. In humans, we found that NSCLC patients bearing high *IL25* gene expression had significantly reduced overall survival as compared to patients with low *IL25* expression. We assessed the frequencies of ILC2 cells in tumor tissue, adjacent normal lung tissue, mediastinal LN and PB of NSCLC patients, as well as in PB from HDs. This is the first time that such a comprehensive study has been done. We found that ILC2 cells were enriched in NSCLC patients compared to HDs, confirming previous results by Shen et al. (8) with a different cohort of patients. In addition to the results provided by Shen et al. (8), we assessed the frequencies of ILC2 cells in lung-draining mediastinal LNs and normal lung tissue adjacent to tumor of NSCLC patients, and found that the increase in ILC2 cells was systemic. As previously reported, ILC2 cells were less represented in LNs than in the other tissues (17). We found similar frequencies of ILC2s in tumor tissue and its normal counterpart. While this result is consistent with the study by Shen et al. (8), it is in contrast with a previous report by Carrega et al. (9). The discrepancy may be explained by the nearly double number of samples assessed in our study. In addition to previous studies, we report absolute numbers of ILC2s per mg of tissue and found that they were significantly higher in tumors as compared to normal lung tissue. Of note, we identified ILC2s by their expression of the surface marker CRTH2. However, Mazzurana et al. (28) reported that a population of CRTH2-negative ILC2 cells potentially representing activated ILC2 cells could be found specifically in the human lung. Our study therefore does not include this population of ILC2 cells.

In addition to ILC2s, we assessed the frequencies of total CD4^+^ T cells and Tregs in the obtained NSCLC and HD samples. We found that total CD4^+^ T cells were reduced while Tregs were enriched in NSCLC patients. Moreover, Tregs accumulated preferentially in tumors and tumor-draining LNs. To the best of our knowledge, this is the first comprehensive report concomitantly comparing Treg and ILC2 frequencies in various tissues of NSCLC patients and HDs by flow cytometry.

While the role of ILC2 cells in allergy and parasitic infections is now well-established, their role in cancer is still controversial (3). In a murine model of heterotopic lung cancer expressing IL-33, ILC2-deficient mice, which were generated by reconstituting their bone marrow with RORalpha^-/-^ hematopoietic stem cells, showed increased tumor growth and metastasis, suggesting an anti-tumor role for IL-33-activated ILC2 cells (10). In other cancer types such as pancreatic cancer, melanoma, lymphoma or colon cancer, IL-33-activated ILC2 cells were reported to bear protective functions (reviewed in [3-4]). However, recent evidence suggests an opposite function for IL-25-activated ILC2 cells. In a mouse model of colorectal cancer, IL-25 activation of ILC2 cells promoted intestinal tumorigenesis by recruiting and activating immunosuppressive M-MDSCs (13). Here, the discrepancy may be explained by three different reasons: first, different cancer types have disparate TMEs, immune compositions and immune reactions. Second, different models of the same cancer type have distinct genetic mutations that can influence the immune environment and therefore the immune reactions to tumors. Finally, differing activation signals received by ILC2 cells may lead to different outcomes. It has recently been proposed that IL-33-responsive ILC2 cells represent ‘natural ILC2s’ (nILC2s) while IL-25-responsive ILC2 cells constitute ‘inflammatory ILC2s’ (iILC2s), both with distinct tissue localization, cell surface marker expression, and potentially distinct functions (5). The presence of different subsets of ILC2 cells in IL-33-expressing and IL-25-expressing cancers could explain the discrepancies in their observed functions.

The ILC2 cells used in our study are consistent with the described iILC2s by Huang et al. (5). Indeed, our ILC2 cells are induced by IL-25, they express high levels of KLRG1, they can be found in the mesenteric LN (MLN) and spleen, and they produce large amounts of IL-13 and IL-4. Our ILC2s migrated to tumors and promoted tumor growth as well as reduced survival in lung tumor-bearing mice, possibly by recruiting M-MDSCs into tumors, consistent with the findings by Jou et al. in CRC (13). This is the first time that IL25-activated ILC2 cells’ role in lung cancer has been studied and our results indicate that such cells bear tumor-promoting functions, in contrast to IL-33-activated ILC2 cells (10). Of note, Huang et al. (29) have reported that iILC2s converted into an nILC2 phenotype after prolonged recombinant IL-25 or IL-33-treatment. Whether this is the case under the physiologic conditions of a tumor remains to be determined. Overall, our study as well as the recent study by Jou et al. (13) strengthen the model of the two distinct subsets of ILC2 cells put forth by Huang et al. (5), and indicate that IL25 may be a novel biomarker with crucial importance for cancer prognosis.

The present study has some limitations. We found that, in mice, M-MDSCs accumulated into tumors following adoptive transfer with IL25-activated ILC2 cells, suggesting that these cells may recruit M-MDSCs. Several reports have shown that IL-13 produced by ILC2 cells can mediate the selective recruitment of M-MDSCs that express IL-13Ralpha1 (25, 26). The ILC2 cells used in our study produce large amounts of IL-13, however we did not show whether the recruitment of M-MDSCs into tumors after ILC2 adoptive transfer was dependent on IL-13 production by ILC2 cells. Further well-designed studies are therefore needed to test for this hypothesis. Moreover, the ILC2 cells used in our study also produce large amounts of IL-4. Jou et al. (13) showed that ILC2-derived IL-4 and IL-13 promoted the suppressive capacities of M-MDSCs by increasing arginase 1 (Arg1) expression, therefore the ILC2 cells used in our study may not only promote the recruitment of M-MDSCs but they may also increase their immunosuppressive capacities. Additional studies, beyond the scope of the present contribution, are needed to better understand the direct effects of ILC2 cells on M-MDSCs. Finally, although we found that ILC2 cells and Tregs were enriched in NSCLC patients, we did not assess the frequencies of M-MDSCs in these patients. Nevertheless, previous studies did report higher frequencies of M-MDSCs both in the peripheral blood and in tumors of NSCLC patients as compared to HDs (30, 31) as well as an association between increased ILC2s and MDSCs in patients with lung cancer (7).

In humans, whether there are two subsets of ILC2 cells, namely IL33-activated or nILC2 and IL25-activated or iILC2 such as in mice, is unknown. Indeed, human ILC2 cells are defined by the expression of CD45, CRTH2 and the lack of expression of lineage markers (1). They also express CD25, ICOS, ST2 (a subunit of IL-33R) and IL-17RB (a subunit of IL-25R) (1). While IL-25 and IL-33 have been shown to induce distinct activation profiles in human ILC2 cells *in vitro* (32), whether IL-25-responsive ILC2s and IL-33-responsive ILC2s constitute different subsets of ILC2 cells in humans is unknown. In NSCLC patients, IL-25R and ST2 expression by ILC2 cells has never been assessed. Although we report CD25 expression in ILC2 cells from NSCLC patients, we did not assess IL-25R nor ST2 expression in our study. Furthermore, due to the rarity of ILC2 cells in clinical samples, we could not assess whether ILC2 cells from NSCLC patients were more responsive to IL-25 or IL-33. At the steady-state, murine lungs are known to bear nILC2 cells that are responsive to IL-33, however, under inflammatory conditions, IL-25-responsive iILC2 cells are quickly induced (5). Whether this is the case in humans as well, and especially in NSCLC patients, remains to be determined.

In summary, our data supports a tumor-promoting role for the IL25/ILC2 axis both in humans and in mice. In mice, IL-25-activated ILC2 cells were able to migrate to tumors, promote tumor growth and metastasis, and reduce the survival of mice. Additionally, they were associated with an accumulation of M-MDSCs in tumors, suggesting that they may actively mediate their recruitment. In humans, high *IL25* expression was associated with reduced survival, and ILC2 cells were found to be enriched in NSCLC patients. Furthermore, concomitant with ILC2 cells, the frequency of immunosuppressive Tregs was also increased. Overall, our results suggest that the IL25/ILC2 axis bears pro-tumoral functions in human and mouse lung cancer, and that targeting it might represent a novel immunotherapy for patients with NSCLC.

## Data availability statement

The original contributions presented in the study are included in the article/**Supplementary Material**. Further inquiries can be directed to the corresponding author.

## Ethics statement

The studies involving humans were approved by Istanbul Medipol University Ethics Committee. The studies were conducted in accordance with the local legislation and institutional requirements. The participants provided their written informed consent to participate in this study. The animal study was approved by Istanbul Medipol University Animal Experiments Local Ethics Committee (IMU-HADYEK). The study was conducted in accordance with the local legislation and institutional requirements.

## Author contributions

Conception, design and supervision: FM. Development of methodology: IB. Acquisition of human material: AT, MG, AÇ, IB. Acquisition of murine data: IB, ZE, OK. Analysis and interpretation of data: IB, FM. Writing and review of the manuscript: FM, DD. All authors contributed to the article and approved the submitted version.

## References

[1] Vivier E Artis D Colonna M Diefenbach A Di Santo JP Eberl G. Innate lymphoid cells: 10 years on. Cell (2018) 174:1054–66. doi: 10.1016/j.cell.2018.07.017 30142344

[2] Cheng H Jin C Wu J Zhu S Liu Y-J Chen J. Guards at the gate: physiological and pathological roles of tissue-resident innate lymphoid cells in the lung. Protein Cell (2017) 8:878–95. doi: 10.1007/s13238-017-0379-5 PMC5712288 28271447

[3] Magnusson FC Bahhar I. Helper innate lymphoid cells as cell therapy for cancer. Immunology (2022) 168:569–79. doi: 10.1111/imm.13599 36288454

[4] Trabanelli S Chevalier MF Derré L Jandus C. The pro- and anti-tumor role of ILC2s. Semin Immunol (2019) 41:101276. doi: 10.1016/j.smim.2019.04.004 31130471

[5] Huang Y Paul WE. Inflammatory group 2 innate lymphoid cells. Int Immunol (2016) 28(1):23–8. doi: 10.1093/intimm/dxv044 26232596 PMC4715228

[6] Siegel RL Miller KD Fuchs HE Jemal A. Cancer statistics, 2022. CA Cancer J Clin (2022) 72:7–33. doi: 10.3322/caac.21708 35020204

[7] Wu Y Yan Y Su Z Bie Q Chen X Barnie PA. Enhanced circulating ILC2s and MDSCs may contribute to ensure maintenance of Th2 predominant in patients with lung cancer. Mol Med Rep (2017) 15:4374–81. doi: 10.3892/mmr.2017.6537 28487978

[8] Shen C Liu C Zhang Z Ping Y Shao J Tian Y. PD-1 affects the immunosuppressive function of group 2 innate lymphoid cells in human non-small cell lung cancer. Front Immunol (2021) 12:680055. doi: 10.3389/fimmu.2021.680055 34194433 PMC8237944

[9] Carrega P Loiacono F Di Carlo E Scaramuccia A Mora M Conte R. NCR+ILC3 concentrate in human lung cancer and associate with intratumoral lymphoid structures. Nat Commun (2015) 6:8280. doi: 10.1038/ncomms9280 26395069

[10] Saranchova I Han J Zaman R Arora H Huang H Fenninger F. Type 2 innate lymphocytes actuate immunity against tumours and limit cancer metastasis. Sci Rep (2018) 8:2924. doi: 10.1038/s41598-018-20608-6 29440650 PMC5811448

[11] Schuijs MJ Png S Richard AC Tsyben A Hamm G Stockis J. ILC2-driven innate immune checkpoint mechanism antagonizes NK cell antimetastatic function in the lung. Nat Immunol (2020) 21:998–1009. doi: 10.1038/s41590-020-0745-y 32747815 PMC7116357

[12] Moral JA Leung J Rojas LA Ruan J Zhao J Sethna Z. ILC2s amplify PD-1 blockade by activating tissue-specific cancer immunity. Nature (2020) 579:130–5. doi: 10.1038/s41586-020-2015-4 PMC7060130 32076273

[13] Jou E Rodriguez-Rodriguez N Ferreira A-CF Jolin HE Clark PA Sawmynaden K. An innate IL-25–ILC2–MDSC axis creates a cancer-permissive microenvironment for Apc mutation–driven intestinal tumorigenesis. Sci Immunol (2022) 7(72):eabn0175. doi: 10.1126/sciimmunol.abn0175 35658010 PMC7612821

[14] Thelen TD Green RM Ziegler SF. Acute blockade of IL-25 in a colitis associated colon cancer model leads to increased tumor burden. Sci Rep (2016) 6:25643. doi: 10.1038/srep25643 27165713 PMC4863374

[15] Huang Q Jacquelot N Preaudet A Hediyeh-zadeh S Souza-Fonseca-Guimaraes F McKenzie ANJ. Type 2 innate lymphoid cells protect against colorectal cancer progression and predict improved patient survival. Cancers (Basel) (2021) 13:559. doi: 10.3390/cancers13030559 33535624 PMC7867134

[16] Frech M Knipfer L Wirtz S Zaiss MM. An in *vivo* gene delivery approach for the isolation of reasonable numbers of type 2 innate lymphoid cells. MethodsX (2020) 7:101054. doi: 10.1016/j.mex.2020.101054 33005569 PMC7509459

[17] Simoni Y Fehlings M Kløverpris HN McGovern N Koo SL Loh CY. Human innate lymphoid cell subsets possess tissue-type based heterogeneity in phenotype and frequency. Immunity (2017) 46:148–61. doi: 10.1016/j.immuni.2016.11.005 PMC7612935 27986455

[18] Hepworth MR Monticelli LA Fung TC Ziegler CGK Grunberg S Sinha R. Innate lymphoid cells regulate CD4+ T-cell responses to intestinal commensal bacteria. Nature (2013) 498:113–7. doi: 10.1038/nature12240 PMC3699860 23698371

[19] Oliphant CJ Hwang YY Walker JA Salimi M Wong SH Brewer JM. MHCII-mediated dialog between group 2 innate lymphoid cells and CD4+ T cells potentiates type 2 immunity and promotes parasitic helminth expulsion. Immunity (2014) 41:283–95. doi: 10.1016/j.immuni.2014.06.016 PMC4148706 25088770

[20] Mirchandani AS Besnard A-G Yip E Scott C Bain CC Cerovic V. Type 2 innate lymphoid cells drive CD4 + Th2 cell responses. J Immunol (2014) 192:2442–8. doi: 10.4049/jimmunol.1300974 24470502

[21] Symowski C Voehringer D. Th2 cell-derived IL-4/IL-13 promote ILC2 accumulation in the lung by ILC2-intrinsic STAT6 signaling in mice. Eur J Immunol (2019) 49:1421–32. doi: 10.1002/eji.201948161 31144294

[22] Jiang M Liu H Li Z Wang J Zhang F Cao K. ILC2s induce adaptive th2-type immunity in acute exacerbation of chronic obstructive pulmonary disease. Mediators Inflammation (2019) 2019:1–12. doi: 10.1155/2019/3140183 PMC6610743 31320835

[23] Taylor S Huang Y Mallett G Stathopoulou C Felizardo TC Sun M-A. PD-1 regulates KLRG1+ group 2 innate lymphoid cells. J Exp Med (2017) 214:1663–78. doi: 10.1084/jem.20161653 PMC5461001 28490441

[24] Spits H Artis D Colonna M Diefenbach A Di Santo JP Eberl G. Innate lymphoid cells — a proposal for uniform nomenclature. Nat Rev Immunol (2013) 13:145–9. doi: 10.1038/nri3365 23348417

[25] Chevalier MF Trabanelli S Racle J Salomé B Cesson V Gharbi D. ILC2-modulated T cell–to-MDSC balance is associated with bladder cancer recurrence. J Clin Invest (2017) 127:2916–29. doi: 10.1172/JCI89717 PMC5531411 28650339

[26] Trabanelli S Chevalier MF Martinez-Usatorre A Gomez-Cadena A Salomé B Lecciso M. Tumour-derived PGD2 and NKp30-B7H6 engagement drives an immunosuppressive ILC2-MDSC axis. Nat Commun (2017) 8:593. doi: 10.1038/s41467-017-00678-2 28928446 PMC5605498

[27] Gabrilovich DI Nagaraj S. Myeloid-derived suppressor cells as regulators of the immune system. Nat Rev Immunol (2009) 9:162–74. doi: 10.1038/nri2506 PMC2828349 19197294

[28] Mazzurana L Czarnewski P Jonsson V Wigge L Ringnér M Williams TC. Tissue-specific transcriptional imprinting and heterogeneity in human innate lymphoid cells revealed by full-length single-cell RNA-sequencing. Cell Res (2021) 31(5):554–68. doi: 10.1038/s41422-020-00445-x PMC8089104 33420427

[29] Huang Y Guo L Qiu J Chen X Hu-Li J Siebenlist U. IL-25-responsive, lineage-negative KLRG1(hi) cells are multipotential 'inflammatory' type 2 innate lymphoid cells. Nat Immunol (2015) 16(2):161–9. doi: 10.1038/ni.3078 PMC4297567 25531830

[30] Zadian SS Adcock IM Salimi B Mortaz E. Circulating levels of monocytic myeloid-derived suppressor cells (M-MDSC) and CXCL-8 in non-small cell lung cancer (NSCLC). Tanaffos (2021) 20:15–21. 34394365 PMC8355929

[31] Yamauchi Y Safi S Blattner C Rathinasamy A Umansky L Juenger S. Circulating and tumor myeloid-derived suppressor cells in resectable non–small cell lung cancer. Am J Respir Crit Care Med (2018) 198:777–87. doi: 10.1164/rccm.201708-1707OC 29617574

[32] Camelo A Rosignoli G Ohne Y Stewart RA Overed-Sayer C Sleeman MA. IL-33, IL-25, and TSLP induce a distinct phenotypic and activation profile in human type 2 innate lymphoid cells. Blood Adv (2017) 1:577–89. doi: 10.1182/bloodadvances.2016002352 PMC5728348 29296700

