# Supplementary material for: The IL-25/ILC2 axis promotes lung cancer with a concomitant accumulation of immune-suppressive cells in tumors in humans and mice
Source: Front Immunol. 2023 Sep 15;14:1244437. doi: 10.3389/fimmu.2023.1244437 (PMC10540623; doi:10.3389/fimmu.2023.1244437)
Supplement: Supplementary file 1 [file DataSheet_1.pdf]

Supplementary Table S1. Clinicopathological characteristics of patients with NSCLC

**Patient characteristics**		**NSCLC patients (n=30) n (%)**
Age	<65	20 (57 %)
	>= 65	10 (29 %)
Gender	Female	5 (14 %)
	Male	25 (71 %)
T Stage	T1	7 (23 %)
	T2	12 (40 %)
	T3	7 (23 %)
	T4	4 (13 %)
N Stage	N0	15 (50 %)
	N1	10 (33 %)
	N2	5 (17 %)
M Stage	M0	29 (97 %)
	Mx	1 (3 %)
	M1	0 (0 %)
Survival	Ex	13 (43 %)
	Alive	17 (57 %)
Mean Overall Survival (months)	Ex	12,54
Smoking Status	Active	15 (50 %)
	Quitted	11 (37 %)
	Non-Smoker	4 (13 %)
