# Supplementary material for: The IL-25/ILC2 axis promotes lung cancer with a concomitant accumulation of immune-suppressive cells in tumors in humans and mice
Source: Front Immunol. 2023 Sep 15;14:1244437. doi: 10.3389/fimmu.2023.1244437 (PMC10540623; doi:10.3389/fimmu.2023.1244437)
Supplement: Supplementary file 2 [file DataSheet_2.pdf]

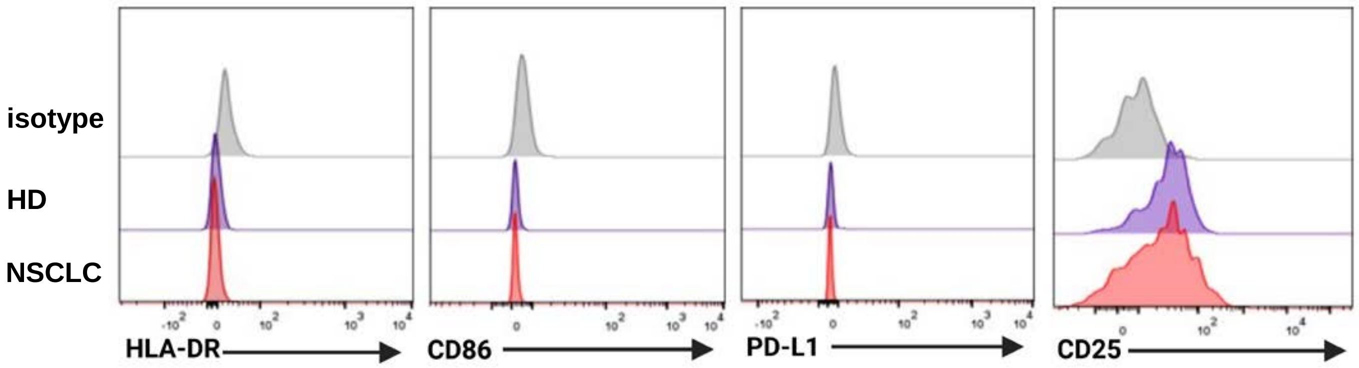

SUPPLEMENTARY FIGURE 1. Phenotypic characterization of ILC2 cells in NSCLC patients and HD. HLA-DR, CD86, PD-L1, and CD25 expression by ILC2 cells determined by flow cytometry analysis of cell surface marker gated on live $CD45^+$ Lin^- $CD127^+$ $CRTH2^+$. Specific antibodies (purple and red lines) or appropriate isotype controls (gray lines) are shown. One representative example out of n=22 (NSCLC) and n=15 (HD) is shown. NSCLC: tumor sample from non-small cell lung cancer patient; HD: peripheral blood sample from healthy donor.

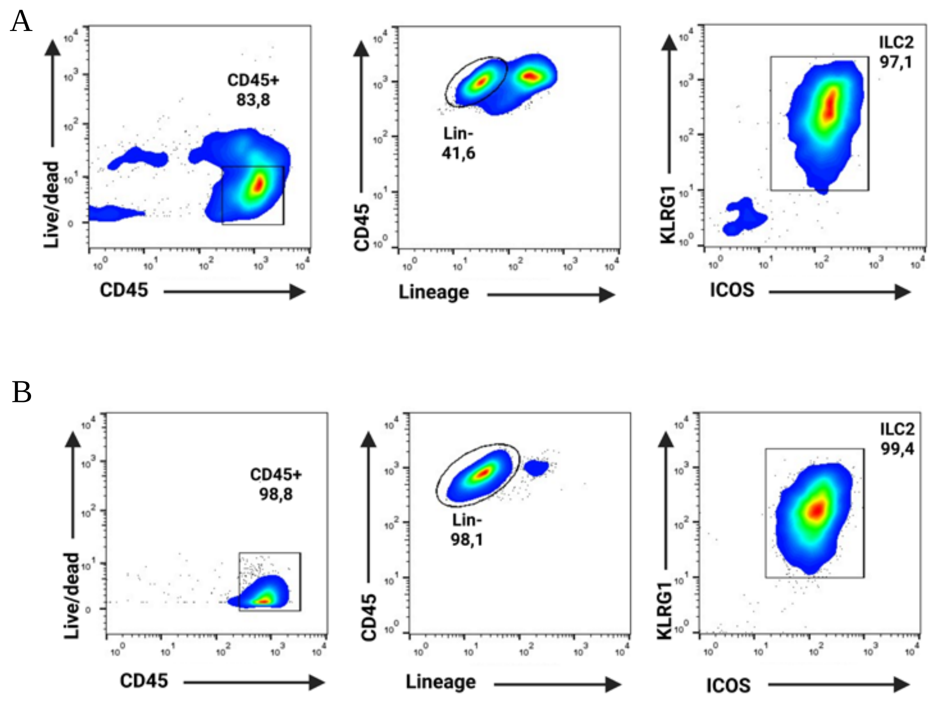

SUPPLEMENTARY FIGURE 2. Cell sorting for in vivo expanded ILC2 cells 3 days after hgd with pCMV-IL25 in mice. (A) Pre-sort purity of ILC2 cells (live CD45$^+$ Lin (CD3, CD5, B220, CD11b, CD11c, NK1.1, TER-119, Gr1, CD170, FcεRIα, CD19, TCRβ, TCRγ/δ)$^-$ KLRG1$^+$ ICOS$^+$) in pooled MLN and spleen cell suspensions that were depleted of lineage positive cells with the mouse direct lineage cell depletion kit. One representative example out of n>20 is shown. (B) Post-sort purity of ILC2 cells (live CD45$^+$ Lin (CD3, CD5, B220, CD11b, CD11c, NK1.1, TER-119, Gr1, CD170, FcεRIα, CD19, TCRβ, TCRγ/δ)$^-$ KLRG1$^+$ ICOS$^+$) in pooled MLN and spleen cell suspensions. One representative example out of n>20 is shown.

A

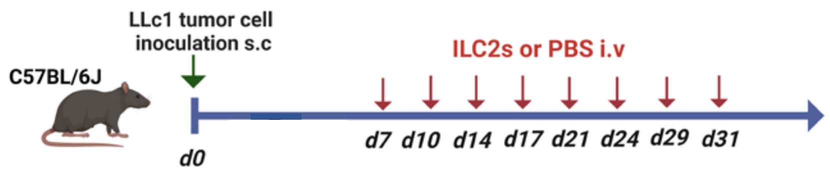

B

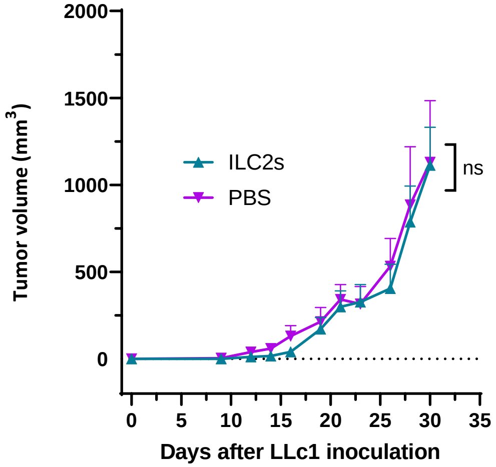

SUPPLEMENTARY FIGURE 3. **Adoptive transfer of ILC2 cells has no effect on tumor growth in non-lymphodepleted recipient mice.** (A) Schematic representation of experimental design. 6-8-week old C56BL/6J mice were inoculated with $1x10^5$ LLc1 lung tumor cells on day 0. Starting on day 7, mice received $5x10^5$ freshly sorted ILC2 cells or vehicle 2 times per week for 4 weeks. s.c: subcutaneous; i.v.: intravenous. (B) Tumor volume in mice that received ILC2s (ILC2s; n=6) vs. mice that received vehicle (PBS; n=8). Data show mean ±sem. ns: not significant (Student t test).

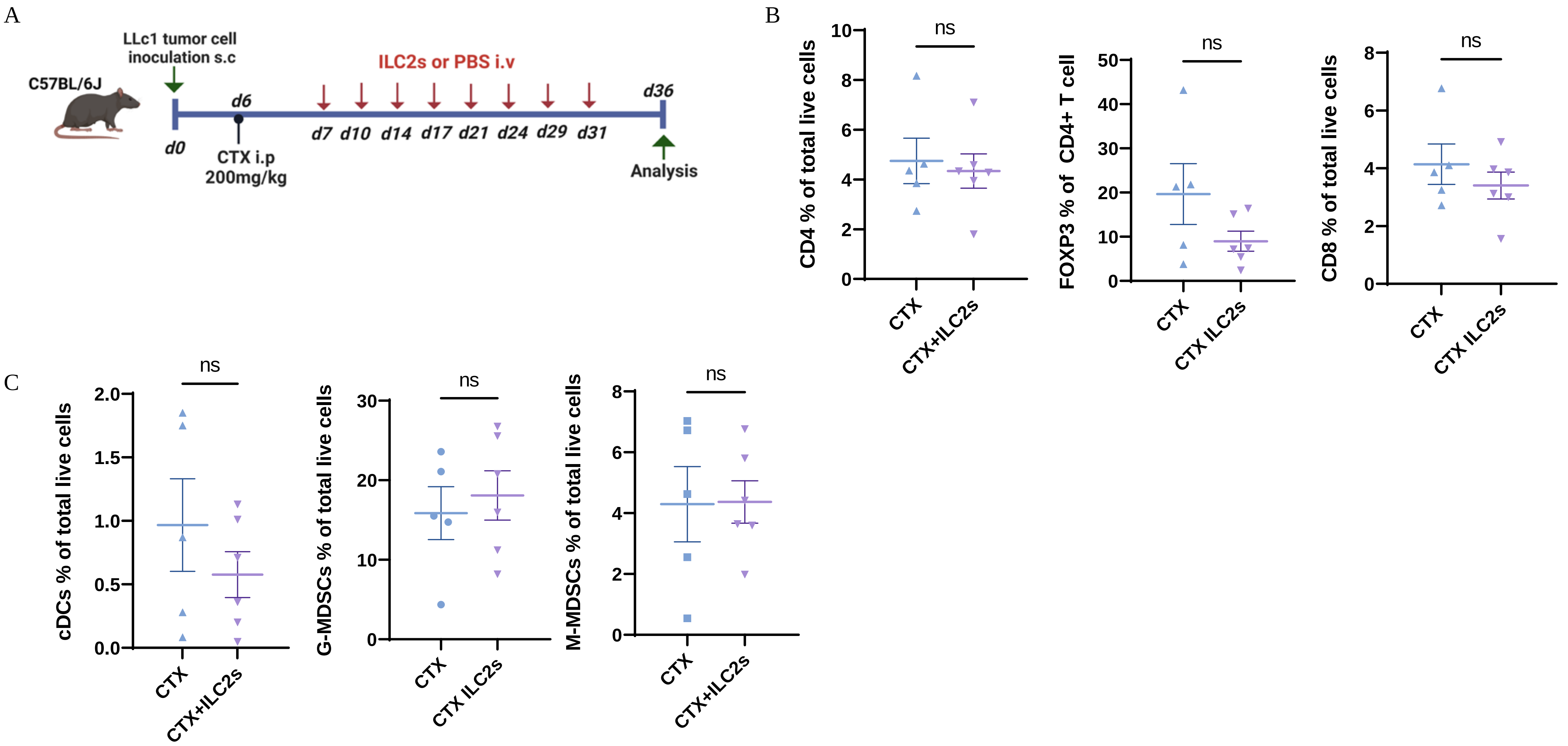

SUPPLEMENTARY FIGURE 4. Immune cell composition in spleens of tumor-bearing mice. (A) Schematic representation of experimental design. 6-8-week old C56BL/6J mice were inoculated with $1x10^5$ LLc1 lung tumor cells on day 0, then partially lymphodepleted with CTX on day 6. Starting one day after CTX administration, mice received $5x10^5$ freshly sorted ILC2 cells or vehicle 2 times per week for 4 weeks. Mice were sacrificed 5 days after the last injection and spleens were collected. s.c: subcutaneous; i.p: intraperitoneal; i.v.: intravenous. (B) Frequency of CD4 T cells identified as $CD3^+$ $CD4^+$, CD8 T cells identified as $CD3^+$ $CD8^+$ and CD4 regulatory T (Treg) cells identified as $CD3^+$ $CD4^+$ $Foxp3^+$ in spleens of vehicle (CTX, n=5)- and ILC2-treated (CTX+ILC2, n=6) tumor-bearing mice. (C) Frequency of conventional DCs (cDCs) identified as $CD11c^+$ $MHCII^+$ cells, granulocytic myeloid-derived suppressor cells (G-MDSCs) identified as $CD3^-$ $CD11b^+$ $Ly6G^+$ $Ly6C^{low}$, and monocytic MDSCs (M-MDSCs) identified as $CD3^-$ $CD11b^+$ $Ly6G^-$ $Ly6C^{hi}$ in spleens of vehicle (CTX, n=5)- and ILC2-treated (CTX+ILC2, n=6) tumor-bearing mice. Horizontal bars mark mean. Error bars mark sem. Statistical significance was calculated by unpaired two-tailed t-test. ns, not significant.
